# Linking MS1 and MS2 signals in positive and negative modes of LC-HRMS in untargeted metabolomics using the ROIMCR approach

**DOI:** 10.1007/s00216-023-04893-3

**Published:** 2023-08-17

**Authors:** Flávia Yoshie Yamamoto, Carlos Pérez-López, Ana Lopez-Antia, Silvia Lacorte, Denis Moledo de Souza Abessa, Romà Tauler

**Affiliations:** 1 Department of Environmental Chemistry, IDAEA-CSIC, Jordi Girona, 18-26, 08034 Barcelona, Spain; 2 Institute of Biosciences, São Paulo State University, São Vicente, Brazil

**Keywords:** Untargeted metabolomics, All-ion fragmentation (AIF), Positive and negative acquisition modes, Metabolite coverage improvement, ROIMCR, Regions of interest multivariate curve resolution

## Abstract

Data-independent acquisition (DIA) mode in liquid chromatography (LC) high-resolution mass spectrometry (HRMS) has emerged as a powerful strategy in untargeted metabolomics for detecting a broad range of metabolites. However, the use of this approach also represents a challenge in the analysis of the large datasets generated. The regions of interest (ROI) multivariate curve resolution (MCR) approach can help in the identification and characterization of unknown metabolites in their mixtures by linking their MS1 and MS2 DIA spectral signals. In this study, it is proposed for the first time the analysis of MS1 and MS2 DIA signals in positive and negative electrospray ionization modes simultaneously to increase the coverage of possible metabolites present in biological systems. In this work, this approach has been tested for the detection and identification of the amino acids present in a standard mixture solution and in fish embryo samples. The ROIMCR analysis allowed for the identification of all amino acids present in the analyzed mixtures in both positive and negative modes. The methodology allowed for the direct linking and correspondence between the MS signals in their different acquisition modes. Overall, this approach confirmed the advantages and possibilities of performing the proposed ROIMCR simultaneous analysis of mass spectrometry signals in their differing acquisition modes in untargeted metabolomics studies.

**Graphical Abstract:**

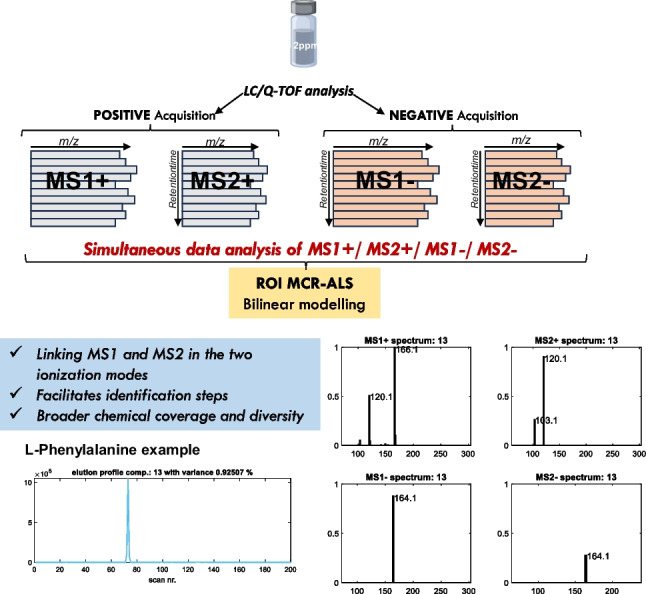

**Supplementary Information:**

The online version contains supplementary material available at 10.1007/s00216-023-04893-3.

## Introduction

New advances in OMICs techniques have increased the comprehension of biological mechanisms as responses to multiple disturbing factors affecting the health of the organisms [1–3]. In this context, the extensive analysis of metabolites represents a powerful approach to unveil new responses of organisms in diverse research fields, such as environmental sciences and clinical studies [4, 5]

Liquid chromatography with high-resolution mass spectrometry (LC-HRMS), either with Orbitrap or time-of-flight (TOF) mass analyzers, represents one of the main choices for the analysis of metabolites in biological systems due to its high sensitivity and specificity [6–8]. While a targeted approach allows the investigation of specific metabolites and potentially affected metabolic pathways, an untargeted analysis assesses a broader range of small molecules, identifying a greater number of affected metabolites and consequently exploring new affected pathways [9].

Regarding the ionization approach, electrospray ionization (ESI) allows the formation of positively or negatively charged ions depending on the polarity of the applied voltage. The particular physiochemical properties of each molecule may determine the most adequate ionization acquisition mode, with some presenting better ionization efficiency (IE) in the positive mode and others in the negative [10]. Most commonly, the acquisition is performed either in positive or negative ESI depending on the electronegativity, basicity, and size of the molecules to be analyzed [11], which may limit the identification of all constituents of biological systems if only one ionization mode is used.

The inherent large-sized complex dataset generated by the LC-HRMS, especially for data-independent acquisition (DIA) mode, is a great challenge for properly interpreting the most relevant changes of treated organisms under an experimental condition. Different computer workflows have been proposed for extracting the significant signal features and their chemical assignation in LC-HRMS datasets [12–16]. The regions of interest multivariate curve resolution (ROIMCR) chemometrics approach is based on the bilinear model intrinsic data structure of the LC–MS data [7, 17–20] and represents an effective strategy for compressing and resolving data without losing relevant information and maintaining instrumental mass accuracy. Firstly, to reduce the dimensionality of the data matrices, the “regions of interest” (ROI) approach considers some parameters, such as signal intensity threshold, mass error tolerance, and a minimum number of detected occurrences [17, 21]. This initial “filtering” of the data selects the most relevant signals (features) and eliminates possible interferences and background noise generated by the equipment and solvents used. Following data compression, the resolution of the elution and spectra profiles of the chemical constituents in the analyzed mixtures can be achieved with the multivariate curve resolution alternating least squares (MCR-ALS) method, as it has been extensively reported [22–25].

Within the untargeted metabolomics approach, our research group has recently shown for the first time the possible simultaneous analysis of MS1 and MS2 DIA raw data obtained by LC/Q-TOF by the ROIMCR approach for the identification of per- and poly-fluoroalkyl substances [20]. This new approach allows an improved linking of MS1 and MS2 DIA spectra of the resolved components by ROIMCR, i.e., linking the MS1 signals of the precursor ions with the MS2 signals of their fragment ions for every chemical compound of the analyzed sample. In this sense, a step forward in this approach is the simultaneous analysis of DIA acquisition in positive and negative modes, which has not yet been exploited. Therefore, in this work, we propose the simultaneous analysis of MS1 and MS2 DIA for both positive and negative modes, to increase the coverage of possible metabolites identified as well as to improve the efficiency of the data analysis procedures in untargeted metabolomics studies.

## Material and methods

First, one synthetic standard mixture of amino acids was prepared and analyzed to set up the ionization and acquisition parameters to obtain higher sensitivity and accuracy by the developed LC/Q-TOF method. Then, 7 fish embryo replicates (*Rhamdia quelen*) were analyzed to comprehensively identify the amino acids’ signatures in the biological samples. For both situations, the simultaneous analysis of MS1 and MS2 DIA datasets for positive and negative modes was attempted using the proposed ROIMCR approach.

### Amino acid analytical standards

The amino acid standard solution containing 17 amino acids and ammonium chloride was purchased from Sigma Aldrich® (AAS18). The concentrated original solution of 2.5 µM mL^−1^ (except by l-cystine at 1.25 µM mL^−1^) was diluted (5 × with 50:50 ACN/water) to reach a concentration of approximately 2 µg/mL (detailed information available in the Supplementary Material). Two internal standards, methionine sulfone (Sigma Aldrich®) and the 1,4-piperazinediethanesulfonic acid (PIPES, Sigma Aldrich®), were prepared at a concentration of 1 mg/mL and were spiked at a final concentration of 1 µg/mL.

### Metabolites' extraction in fish embryo samples

For the analysis of amino acids in biological samples, fish embryos (*Rhamdia quelen*) were obtained in the Panama Fish Farm (Paulo Lopes, Brazil) in March 2021 using the same procedures for fish breeding as described by Yamamoto et al. [26]. Amino acids and other metabolites were extracted from 7 pooled samples (approximately 50 mg of lyophilized embryos for each sample) according to the method proposed by Ortiz-Villanueva et al. [27] and are fully detailed in the Supporting Information.

### LC/Q-TOF analysis

Analysis was performed using a UPLC/Q-TOF (Bruker Impact II). An Acquity UPLC BEH HILIC column (2.1 × 50 mm, 1.7 μm) from Waters™ was used to analyze amino acids and other metabolites in the standard solution and fish embryo extracted samples according to the protocol proposed by WATERS [28] with few modifications (Table S1 in Supporting Information). Acquisition (FSR > 60,000) was performed in full scan mass over 60 to 1000 m/z (Supporting Information Table S2). The ionization source operated in positive (4 eV of ion energy, 20–30 eV switching normalized collision energy) and negative mode (6 eV of ion energy, 24–36 eV switching normalized collision energy) alternating MS scans of the precursor ions (MS1) and all-ion fragmentation (MS2). Acquisitions in positive and negative ESI were performed in different chromatographic runs, as Bruker’s Q-TOF technology does not allow simultaneous acquisition in both modes. All solvents were analytical grade or higher purity.

### Data analysis

**Fig. 1:**
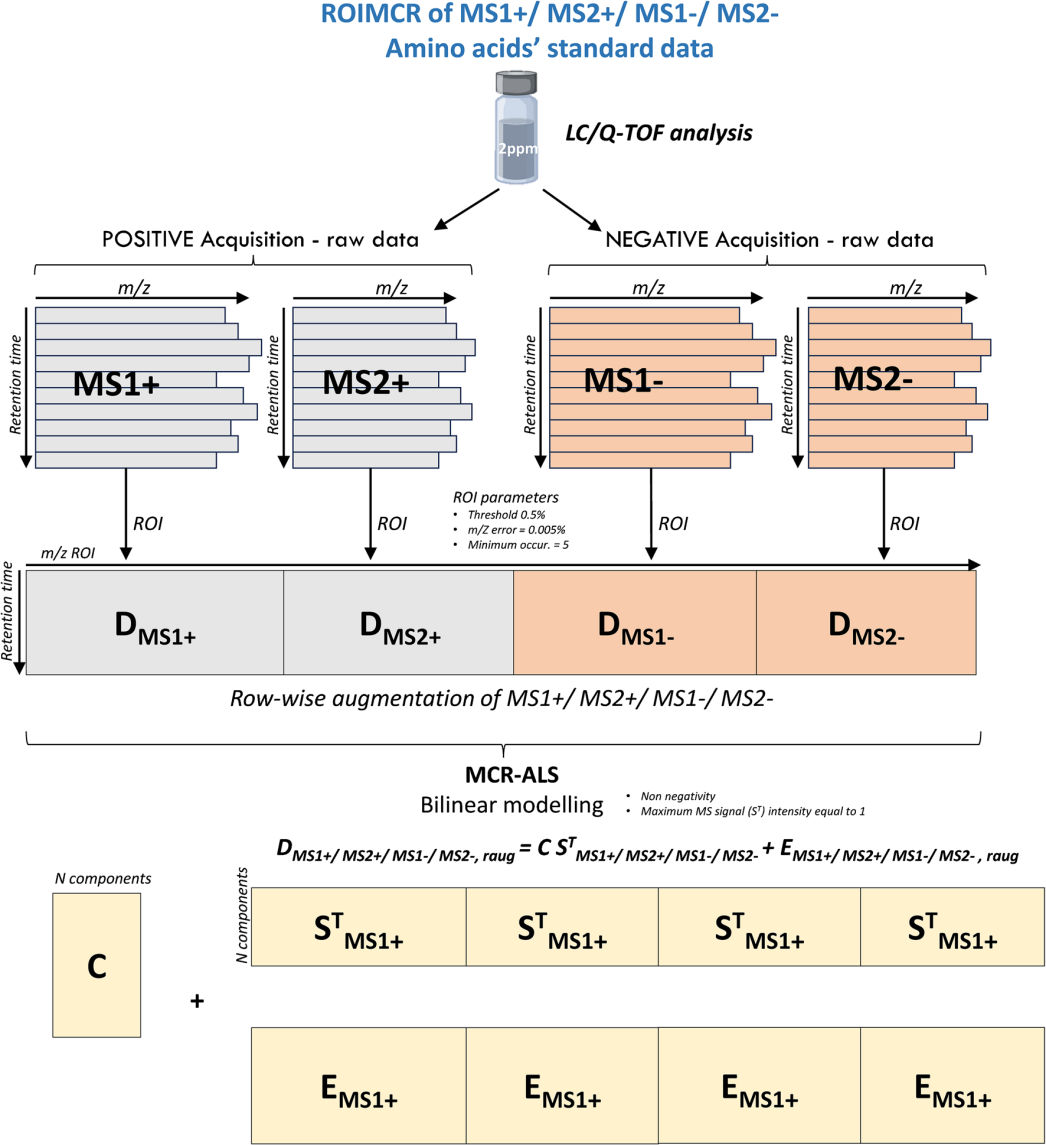
Row-wise matrix augmentation and MCR bilinear model factor decomposition of MS1( +), MS2( +), MS1(-), and MS2(-) data acquired in the analysis of the amino acid standard mixture

LC/Q-TOF data obtained in the analysis of the amino acid standards and fish embryo samples were independently processed by the ROIMCR chemometric method [17, 22, 24, 29], which consists of a first step (regions of interest, ROI) to select the most relevant MS instrumental signals reducing the dimensionality of the data [21] followed by the application of the multivariate curve resolution (MCR) method according to Jaumot et al. [18] and Tauler [30] to resolve the elution and mass spectra profiles of the chemical constituents of the analyzed samples. In this work, MS1 and MS2 data acquired independently (DIA) were simultaneously analyzed by ROIMCR either separately in positive ( +) and negative (-) ionization modes or simultaneously, in MS1( +), MS1(-), MS2( +), and MS2(-) modes. The procedure has the following steps (Fig. 1).

#### Import of data

Firstly, the raw data obtained through the Bruker LC/Q-TOF were converted to a NetCDF data file (or mzXML files) through the Compass DataAnalysis software 5.2 (Bruker, Germany) and imported into MATLAB R2020a computing and visualization environment. This could be performed either using the MSroi app as described by Pérez-Cova et al. [21] in MATLAB or via mass spectrometry directly with the functions of the Bioinformatics toolbox (The Mathworks, Inc., 2020b).

The data from the amino acids’ standard were imported using the MSroi GUI app as a single chromatographic run (single sample option) while the data from the 7 replicate fish embryo samples were imported as multiple chromatographic runs (using the multi-sample option) arranged as a column-wise augmented data matrix (see Fig. 1).

#### Regions of interest (ROI)

Before applying the ROI compression procedure to the NetCDF imported datasets, the MS1 and MS2 scans in positive ( +) and negative (-) modes were separated, either for the analysis of the single sample (amino acid standards) or for the simultaneous analysis of the multiple fish embryo samples. After the data separation, ROI compression was performed separately for MS1( +), MS1(-), MS2( +), and MS2(-) datasets. Additionally, small parts of the chromatograms were cut by selecting only the time intervals of interest, disregarding the first 0.3 min (corresponding to the column dead volume) of the chromatographic run.

The ROI analysis generates a data matrix with a number of rows equal to the number of elution times and a number of columns equal to the number of MSROIs. This data matrix has the filtered MSROI data (following the parameters previously mentioned for each MS1 and MS2 dataset). The data matrices resulting from the ROI compression can be augmented row-wisely (horizontal concatenation) and be analyzed simultaneously in the two acquisition modes, the MS1 and the MS2 DIA data, at the same chromatographic retention times. This row-wise data matrix augmentation can be extended to include MS1 and MS2 DIA modes in both positive and negative ionization modes. An additional step for filtering those MSROIs with good elution profiles (through graphical visualization) was performed to improve the analysis before performing multivariate curve resolution, excluding those MSROIs with poor resolution, usually related to noise, also present in blank samples.

It is important to highlight that the row-wise matrix augmentation to analyze MS1 and MS2 DIA in positive and negative ionization modes requires that they are aligned to the same retention times. In other words, even if the acquisitions were performed in different chromatographic runs (as it occurred in the current study), the retention times between these datasets should be aligned as much as possible before the horizontal concatenation of the corresponding individual data matrices in the different acquisition modes. Therefore, MS signals were interpolated to match the chromatographic retention times of the two acquisition modes before the horizontal concatenation of positive and negative MS signals.

#### Multivariate curve resolution-alternating least squares (MCR-ALS)

1 $${\mathbf D}_{\mathbf M\mathbf S}=\mathbf C\boldsymbol\;\mathbf S_{\mathbf M\mathbf S}^{\mathbf T}+\mathbf E$$

Previously arranged MSROI data matrices, **D**_**MS**_, were decomposed according to a factor decomposition bilinear model using MCR-ALS [18, 30] into the product of two-factor matrices with the elution profiles, **C**, giving the relative concentrations of the compounds, and the MS spectra profiles, **S**^**T**^, giving the spectra with the MS signals and their m/z values, for the *N* selected components. Not explained variance is stored in the residuals/error data matrix, **E** (Eq. 1).

Components’ spectra normalization was carried out as a constraint by MCR-ALS (spectra equal height) during the resolution of the components of the system. The mass spectra of every resolved component (potentially representing a chemical compound) have the MS1 and MS2 signals of the precursor and fragment ions, respectively, for each acquisition and ionization mode.

MCR can be performed separately for each chromatographic run using one acquisition and ionization mode or simultaneously using multiple chromatographic runs and acquisition, as described by Pérez-López et al. [20], and ionization positive and negative modes, as shown in the following Eqs. 2, 3, 4, and 5.

In the individual MCR-ALS analysis of a single chromatographic run by DIA MS1 and MS2 (Eq. 2), the horizontally concatenated data matrix **D**_**MS1,MS2**_ is decomposed in the product of two-factor matrices, **C** and **S**^**T**^_**MS1,MS2**_, related respectively with the concentration (elution) and with the horizontally concatenated (linked) MS1 and MS2 profiles of the chemical constituents present in the analyzed samples.

2 $${\mathbf{D}}_{\mathbf{M}\mathbf{S}1,\mathbf{M}\mathbf{S}2}=\mathbf{C}{\mathbf{S}}_{\mathbf{M}\mathbf{S}1,\mathbf{M}\mathbf{S}2}^{\mathbf{T}}+{\mathbf{E}}_{\mathbf{M}\mathbf{S}1,\mathbf{M}\mathbf{S}2}$$

When multiple chromatographic runs are analyzed simultaneously, the different individual data matrices, **D**_**MS**_, can be concatenated vertically sharing the same spectral vector space, as shown in Eq. 3 for the case of concatenating chromatographic multi-run data matrices acquired in one of the two acquisition modes, either in positive or negative ionization.

3 $${\mathbf{D}}_{\mathbf{M}\mathbf{S}1,\mathbf{M}\mathbf{S}2,\mathbf{a}\mathbf{u}\mathbf{g}}=\left[\begin{array}{c}{\mathbf{D}}_{\mathbf{M}\mathbf{S}1,\mathbf{M}\mathbf{S}2}\\ {\mathbf{D}}_{\mathbf{M}\mathbf{S}1,\mathbf{M}\mathbf{S},2}\\ \cdots \\ {\mathbf{D}}_{\mathbf{M}\mathbf{S}1,\mathbf{M}\mathbf{S}2,\mathbf{K}}\end{array}\right]=\left[\begin{array}{c}{\mathbf{C}}_{1}\\ {\mathbf{C}}_{2}\\ \cdots \\ {\mathbf{C}}_{\mathbf{K}}\end{array}\right]{\mathbf{S}}_{\mathbf{M}\mathbf{S}1,\mathbf{M}\mathbf{S}2}^{\mathbf{T}}\left[\begin{array}{c}{\mathbf{E}}_{1}\\ {\mathbf{E}}_{2}\\ \cdots \\ {\mathbf{E}}_{\mathbf{K}}\end{array}\right]={\mathbf{C}}_{\mathbf{c}\mathbf{a}\mathbf{u}\mathbf{g}}{\mathbf{S}}_{\mathbf{M}\mathbf{S}1,\mathbf{M}\mathbf{S}2}^{\mathbf{T}}+{\mathbf{E}}_{\mathbf{M}\mathbf{S}1,\mathbf{M}\mathbf{S}2,\mathbf{a}\mathbf{u}\mathbf{g}}$$

where now ${\mathbf{D}}_{\mathbf{M}\mathbf{S}1,\mathbf{M}\mathbf{S}2,\mathbf{a}\mathbf{u}\mathbf{g}}$, **C**_**aug**_, and ${\mathbf{E}}_{\mathbf{M}\mathbf{S}1,\mathbf{M}\mathbf{S}2,\mathbf{a}\mathbf{u}\mathbf{g}}$ refer to the augmented vertically concatenated, concentration (elution profiles), and residual/error matrices, when multiple MSROI data matrices coming from different chromatographic runs are simultaneously analyzed. And **S**^**T**^_**MS1, MS2**_ has as before the horizontally concatenated (linked) MS1 and MS2 profiles of the chemical constituents present in the analyzed samples.

These horizontal and vertical concatenations and MCR-ALS analysis can be also extended (introduced for the first time in this work) to the two possible ionization modes as shown in the following Eqs. 4 and 5

4 $${\mathbf D}_{\mathbf M\mathbf S1+,\mathbf M\mathbf S1-\mathbf M\mathbf S2-,\mathbf M\mathbf S2-}=\mathbf C\boldsymbol\;\mathbf S_{\mathbf M\mathbf S1+,\mathbf M\mathbf S1-,\mathbf M\mathbf S2-,\mathbf M\mathbf S2-}^{\mathbf T}+{\mathbf E}_{\mathbf M\mathbf S1+,\mathbf M\mathbf S2+,\mathbf M\mathbf S1-,\mathbf M\mathbf S2-}$$

5 $${\mathbf{D}}_{\mathbf{M}\mathbf{S}1+,\mathbf{M}\mathbf{S}1+\mathbf{M}\mathbf{S}2-,\mathbf{M}\mathbf{S}2-,\mathbf{a}\mathbf{u}\mathbf{g}}={\mathbf{C}}_{\mathbf{a}\mathbf{u}\mathbf{g}}\boldsymbol{ }{\mathbf{S}}_{\mathbf{M}\mathbf{S}1+,\boldsymbol{ }\mathbf{M}\mathbf{S}1-,\mathbf{M}\mathbf{S}2-,\boldsymbol{ }\mathbf{M}\mathbf{S}2-}^{\mathbf{T}}+{\mathbf{E}}_{\mathbf{M}\mathbf{S}1+,\boldsymbol{ }\mathbf{M}\mathbf{S}2+,\mathbf{M}\mathbf{S}1-,\boldsymbol{ }\mathbf{M}\mathbf{S}2-,\boldsymbol{ }\mathbf{a}\mathbf{u}\mathbf{g}}$$

In all these two equations, the **C** and **C**_**aug**_ concentration matrices, have the elution profiles of every component resolved by MCR-ALS in one of or in multiple chromatographic runs. From the peak areas or heights of the elution profiles resolved in **C** or **C**_**aug**_, relative quantitative information on every resolved component can be obtained. This is especially useful in the case of multiple samples (chromatographic runs) simultaneously analyzed (in **C**_**aug**_), for calibration purposes or semiquantitative estimations, in case of the absence of standards for quantification. With this information, the variation of the concentrations of the metabolites among different samples can be studied.

On the other hand, from the ion signals in the resolved mass spectra, **S**^**T**^_**MS1+,MS1-, MS2-,MS2**_ horizontally concatenated and linked, the compound annotation or identification step is improved.

The complete data analysis workflow and the different types of data matrices concatenations and augmentations are displayed in Figs. 1 (augmentation of multiple acquisition and ionization modes of the amino acids’ standard sample) and 2 (augmentation of multiple chromatographic runs at different acquisition and ionization modes for the fish embryo replicate samples).

**Fig. 2:**
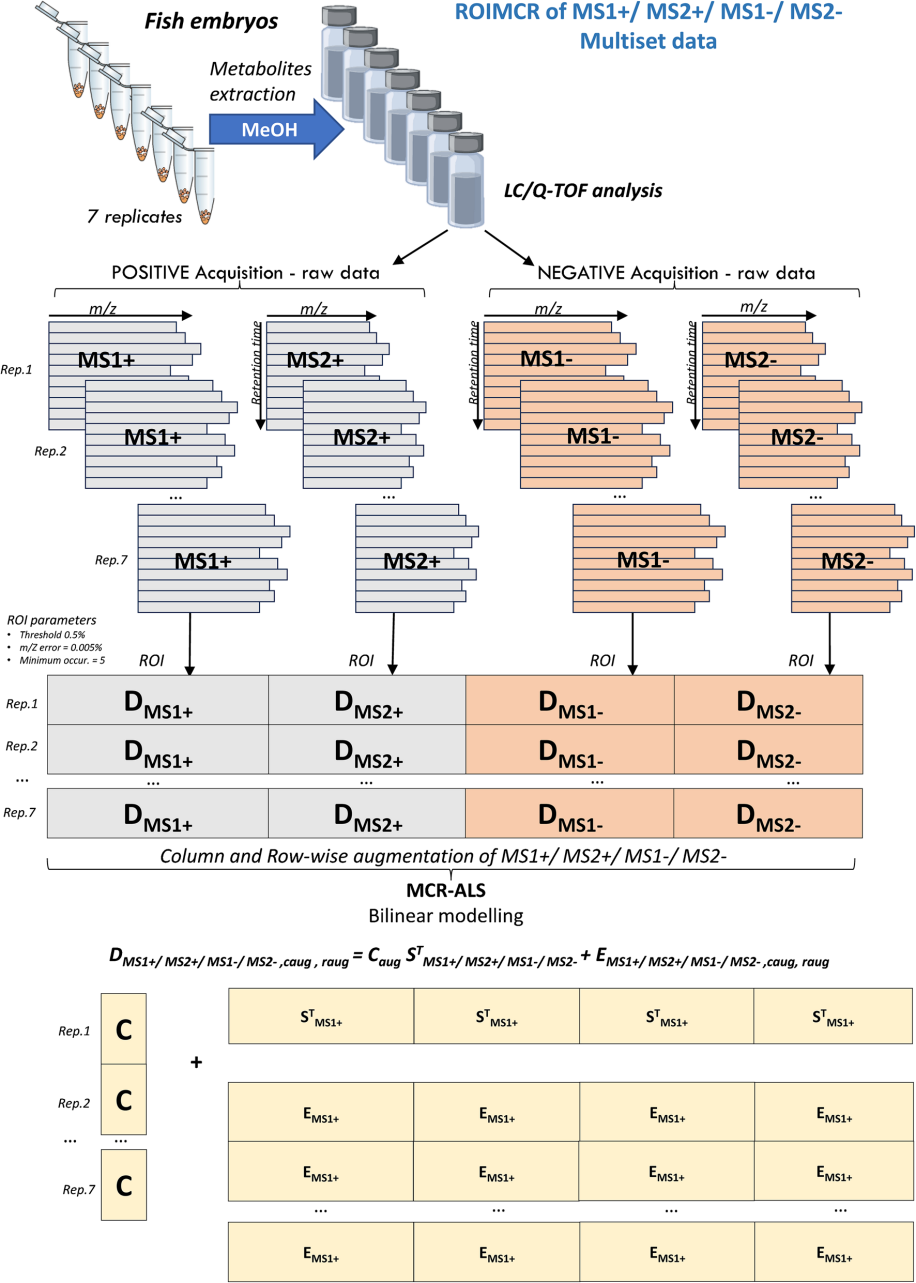
Column- and row-wise matrix augmentation and MCR bilinear model factor decomposition of MS1( +), MS2( +), MS1(-), and MS2(-) data acquired in the analysis of multiple fish embryo sample replicates

The steps of the MCR-ALS analysis of the different MSROI data matrices are also given in the Supporting Information and have been described in previous papers [17, 18, 20, 22, 30].

#### Identification of the amino acids

The identification of the amino acids from the mass spectra profiles resolved by the MCR-ALS, both in the standard solution and in the fish embryo samples, was initially performed by directly checking if their m/z ROI values for precursor (MS1) and fragment (MS2) ions present in the MCR-ALS resolved mass spectra, **S**^**T**^_**MS1,MS2**_, were available in public databases (Mass Bank, PubChem [31], accessed Nov-Dec/2022). To confirm the amino acid identity, a minimum mass error of 0.01 Da both for MS1 and MS2 values was considered. Mass measurement accuracy (average of individual mass errors) of 0.0017 Da was obtained for the positive acquisition mode and 0.0034 Da for the negative acquisition mode, and 0.0025 Da for the simultaneous analysis.

In addition, the identity of the amino acids verified by the ROIMCR approach was further confirmed using the Data Analysis from Bruker’s MS software, and also the MS-DIAL software [14] by checking the reference-matched (MS1 and MS2 spectra were confirmed with literature databases if a total score > 850 in compound search information was found) and suggested ions (only MS1 spectra is confirmed). To do this, the raw MS data NetCDF files were converted to ABF format and the parameters were set with similar conditions to those used for the ROI analysis, with few modifications (intensity threshold of 1000, mass error of 0.01 Da, and other details given in the Table S3 in the Supporting Information) to increase the sensitivity of the analysis and consider more potential compounds, with milder filtering conditions.

## Results and discussion

### ROIMCR results and parameter selection

Significant reduction of the data dimensionality of the amino acid standard sample was achieved by selecting the following ROI parameters (Table 1): a signal intensity threshold of 5000 (the equivalent of 0.1% of maximum intensity), a mass error tolerance of 0.005 Da, and minimum occurrence of 5 scans. Similar parameters were used for the ROI analysis of fish embryo samples, except for the intensity threshold which was set to a higher value of 10,000 (0.5%) due to the increased size of the data for biological samples.

**Table 1:** Regions of interest (ROI) parameters

Regions of interest (ROI) algorithm parameters		
	Aa standard mix	Fish embryo samples
Signal intensity threshold	5000 (~ 0.1%)	10,000 (~ 0.5%)
Factor signal (× threshold)	1	1
Mass error deviation	0.005	0.005
Mass units	Daltons	Daltons
m/z values calculation	Median	Median
Minimum occurrence	5	5

For the analysis of the amino acids’ standards, 30 components were resolved by MCR-ALS (Supporting Table S4), while for the fish embryo samples, 100 components were resolved considering the elevated variety of features present in biological samples. Non-negativity constraints were applied to the elution and mass spectra profiles. All MCR-ALS analyses provided more than 99.5% of explained variance for both the amino acid standard and the fish embryo samples (see Supporting Table S4).

### ROIMCR results in the analysis of the amino acid standard

The LC/Q-TOF method with the HILIC column used in this study represented a fast, simple, and effective strategy for separating and detecting the amino acids (Supporting Figure S1), as reported by [32]. In addition, the ROIMCR approach solves the mixture analysis problem even in situations of strong compounds’ coelutions, by improving the resolution of their elution profiles. Due to the selectivity of high-resolution MS signals, ROIMCR can separate the elution and spectra profiles of a large number of possible chemical constituents in complex biological and environmental samples [7, 19, 27, 29].

ROIMCR analysis allowed identifying all seventeen amino acids present in the standard mixture sample (see Table 2 and Supporting Figure S2), as well as the two internal standards present in the sample. As expected, the most intense signals were observed at higher m/z values for MS1 when compared to MS2 for both acquisition modes.

**Table 2:** Parameters used for the detection of the seventeen amino acid standard solution and the two additional internal standards analyzed. Exact^1^ and experimental^2^ exact mass and their maximum height signal intensity values (arbitrary units)^3^ obtained at each ionization mode (positive and negative), and their chromatographic retention times^4^. The last column^5^ confirms (x) the detection of the amino acid (or internal standard) in the simultaneous data analysis of the positive and negative modes

Amino acids		Positive ionization			Negative ionization				
		[H^+^] exact mass^1^	[H^+^] exper. Mass^2^	Intensity^3^	[H^−^] exact mass^1^	[H^−^] exper. mass^2^	Intensity^3^	RT^4^ (min)	Pos/neg^5^
1	l-Phenylalanine	166.0868	166.0846	**1,091,678**	164.0712	164.0671	584,228	2.82	x
2	l-Isoleucine	132.1025	132.1007	**1,562,956**	130.0868	130.0837	223,230	2.89	x
3	l-Leucine	132.1025	132.1007	**1,562,956**	130.0868	130.0837	223,230	2.89	x
4	l-Tyrosine	182.0817	182.0793	441,574	180.0661	180.0616	**677,328**	2.99	x
5	l-Methionine	150.0589	150.0569	**946,572**	148.0432	148.0395	816,872	3.03	x
6	l-Valine	118.0868	118.0853	**775,806**	116.0712	ND	ND	3.24	x
7	*Methionine sulfone*	182.0487	182.0463	120,358	180.03305	180.0284	**808,366**	3.34	x
8	Glycine	76.03986	ND	ND	74.0242	74.0223	**286,062**	3.58	x
9	l-Threonine	120.0661	120.0645	95,528	118.0504	118.0474	**919,562**	3.58	x
10	*PIPES*	303.0684	303.0643	82,214	301.05281	301.0448	**611,436**	3.58	x
11	l-Proline	116.0712	116.0697	**4,609,182**	114.0555	ND	ND	3.61	x
12	l-Serine	106.0504	106.0492	38,116	104.0348	104.0322	**813,598**	3.72	x
14	l-Alanine	90.05551	90.0543	**70,432**	88.0399	88.0375	16,284	5.49	x
13	l-Glutamic acid	148.061	148.0592	96,378	146.0453	146.0415	**617,628**	4.00	x
15	l-Aspartic acid	134.0453	134.0438	8814	132.0297	132.0262	**57,966**	5.49	x
16	l-Arginine	175.1195	175.1174	**2,055,464**	173.1039	173.0992	195,606	6.14	x
17	l-Lysine	147.1134	147.1116	**420,304**	145.0977	145.0939	37,522	6.39	x
18	Cystine	241.0316	ND	ND	239.0160	239.0098	**8722**	6.49	*
19	l-Histidine	156.0773	156.0752	**146,004**	154.0616	154.0577	129,352	7.08	x

*ND*, non-detected amino acid in one mode of acquisition

^*^Non-detected amino acid due to its poor ionization in both modes of acquisition under tested conditions

Italic font - Metabolites used as internal standards

Bold values - Highest intensity values for one of the acquisition modes

Different signal intensities were obtained for the acquisitions in positive and negative modes for the different amino acids. When MS1 and MS2 DIA data in positive and negative ionization modes were analyzed separately (Eq. 2), distinct sets of fifteen amino acids were detected, with the l-proline and l-valine being detected only in the positive mode and the l-glycine and l-cystine only in the negative mode. In general, better ionization efficiency (higher intensity values) was obtained for the amino acids detected at the positive mode (average signal intensities of 928 and 117 units with 10 amino acids with the highest intensities, Table 2), than at the negative mode (average of signal intensities of 395and 887). This is also illustrated in Supporting Figure S2 where higher peaks are observed for the elution profiles in positive mode. On the other hand, both internal standards showed higher signal intensities in negative mode.

According to the literature, the ionization of amino acids is mostly operated in the positive mode due to their higher ionization efficiency [11, 32, 33]. But alternatively, in some cases, it gives also good results in negative acquisition [34]. Liigand et al. [10] reported better ionization energy in the negative mode for 33 compounds that ionize in both modes, including amino acids. The amino acids’ inherent amphoteric property allows functional groups to either receive or donate protons, therefore having a zwitterionic form. Indeed, their different physiochemical properties determine their different ionization efficiencies [32]. When MS1 and MS2 DIA data in both positive and negative ionization modes were simultaneously analyzed (Eq. 4) by the ROIMCR, all amino acids (except for cysteine due to its poor ionization in both modes under tested conditions) present in the amino acid standard mixture were properly detected and resolved.

Even so, this evidences the relevance and advantages of this method, since for instance, the single analysis in positive mode could have missed the detection of the glycine, while in negative mode could have missed the identification of valine and proline.

**Fig. 3:**
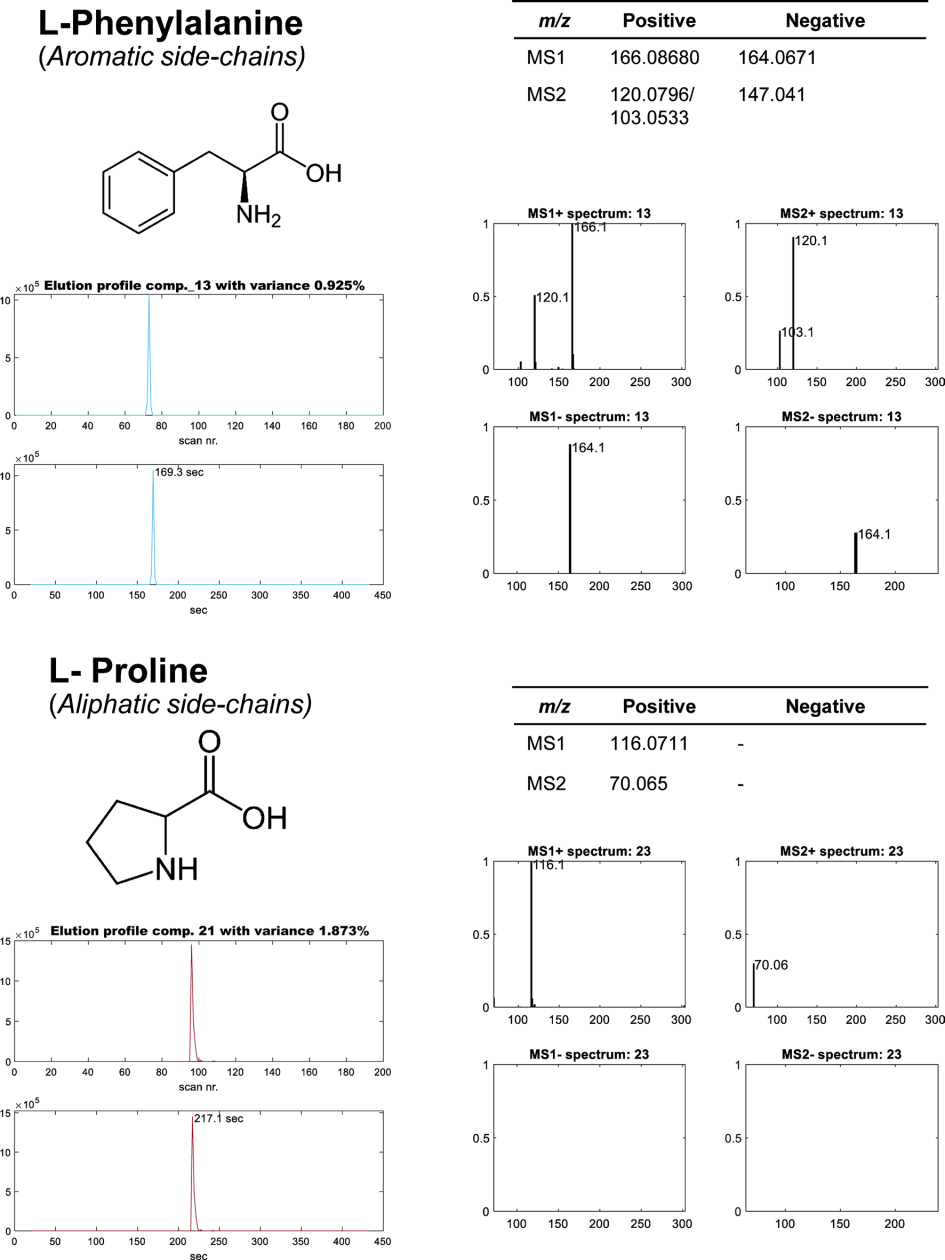
Elution and spectra profiles of two different amino acids (l-phenylalanine and l-proline) resolved by ROIMCR simultaneous analysis of MS and MS/MS DIA data in positive and negative acquisition modes. Left panels: molecular formula of the identified amino acid (upper), ROIMCR elution profiles of the amino acid vs scan number (middle), and vs retention time (lower). Right panels: list of m/z values with more significative MS1 and MS2 signals (upper), and MS1( +), MS2( +), MS1(-), and MS2(-) ROIMCR resolved spectra profiles with the m/z values of their more intense signals (lower)

Figure 3 gives, as an example, the elution and spectra profiles of two different amino acids (l-phenylalanine and l-proline) detected in MS1 and MS2 DIA in simultaneous data analysis of positive and negative acquisition modes (TIC, total ion chromatograms are shown in Figure S1 in the Supporting Information). Apart from their molecular formulas, MS1 and MS2 major ions in positive and negative modes obtained from their respective ROIMCR resolved spectra in the four modes, MS1( +)/MS2( +)/MS1(-)/MS2(-), are given, as well as their elution profiles plotted for the number of scans or with respect to the retention times in the *x*-axis. In this case, as expected, l-phenylalanine elutes earlier (169.3 s) than l-proline (217.1 s). This is explained by the lower polarity of the former when compared with the latter. For both amino acids, higher ionization efficiencies were observed in the positive acquisition mode as reflected in the different scales of the *y*-axis of the resolved mass spectra. In the four cases (MS1( +), MS2( +), MS1(-), MS2(-) signals), their values were scaled to their maximum signal intensity value equal to 1, which was achieved for MS1 in positive mode for both l-phenylalanine and l-proline. In fact, the latter did not give a significant MS- signal. The annotation of the different amino acids can be potentially enhanced using the correspondent link between the main precursor ions in positive (protonated ion [H^+^]) and negative (deprotonated ion [H^−^]) modes, differing approximately 2.01 m/z units. Thus, the proposed ROIMCR allows the direct linking and correspondence between the MS signals in the four acquisition modes (MS1( +)/MS2( +)/MS1(-)/MS2(-)), for the same chemical species (see Tables 2 and S5), when performing their simultaneous analysis. Linking the spectra of the different amino acids in their four acquisition modes facilitates their coverage, identification, and possible quantitation [20]. Table S5 (in Supplementary Information) describes the list of MS2 fragment ions obtained in the ROIMCR analysis, confirmed by the literature database, that facilitated the annotation and identification of the amino acids in the standard sample.

### ROIMCR results in the analysis of the fish embryo samples

After confirming the detection of the amino acids in their standard mixtures, their presence in the fish embryo samples was analyzed using the ROIMCR approach. Similarly to the pattern observed for the amino acids’ standard, it was possible to observe a better ionization efficiency in the positive mode, with more ROI signals of higher intensity being obtained in this acquisition mode when compared to the negative mode (Table 2, Supporting Figure S3). All seven replicates in each acquisition mode gave relatively similar elution profiles. Higher intensity signals were obtained for MS1 ions in comparison to MS2 ion fragments.

**Fig. 4:**
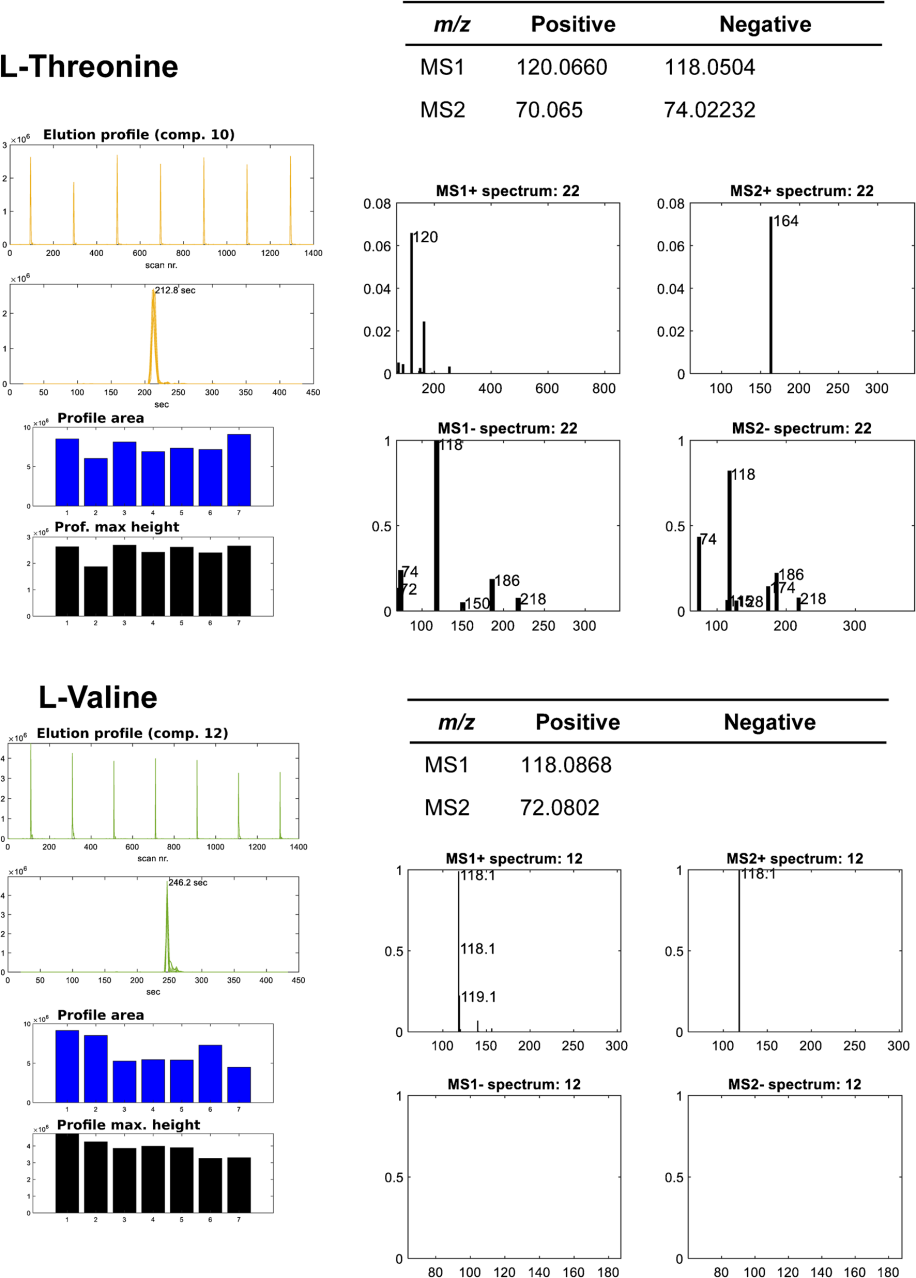
Elution and spectra profiles of two different amino acids (l-threonine and l-valine) detected in the fish embryo samples, resolved by the ROIMCR simultaneous analysis of MS1 and MS2 DIA data in positive and negative acquisition modes. Left panels: ROIMCR elution profiles of the amino acid vs scan number (upper), vs retention time (middle), and their peak areas and maximum heights for each sample analyzed. Right panels: MS1 and MS2 signals (upper) and MS1( +), MS2( +), MS1(-), and MS2(-) (lower) signals of the ROIMCR resolved spectra profiles giving the m/z values of their more intense signals

In Fig. 4, ROIMCR results are shown for the resolution of l-threonine (up) and l-valine (down) amino acids in the analysis of the seven different embryo samples. As in Fig. 3, m/z values of the major MS signals for the two amino acids are given for the different acquisition and ionization modes, which agree with those expected for these two amino acids (see Table 2). Whereas l-threonine gave significant signals at the four acquisition and ionization modes, l-valine was giving MS signal only in positive ionization, for both MS1 and MS2 DIA acquisition modes.

In the upper subplot for l-threonine and the lower subplot for l-valine, the seven elution profiles obtained by ROIMCR in the resolution of the seven embryo replicate samples are shown giving good peak shapes. The reproducibility of these peaks indicates the adequacy of the analytical procedure and the correct ROIMCR resolution of these two amino acids in the different fish embryo samples analyzed with only some small differences in the height and peak areas (also in the two subplots below). In the overlayed elution profiles plot also given in the two subplots, proper peak alignment and shapes among the elution profiles of both amino acids were achieved. Finally, on the right side of the two subplots, the spectra profiles of the amino acid show the MS1 and MS2 DIA signals for the two ionization modes. Whereas for l-threonine, MS1 and MS2 DIA signals were recorded in the four acquisition and ionization modes, and no MS signal was obtained for valine in the negative mode due to its poorer ionization at the conditions of this work.

In traditional analytical work, a broad assessment of multiple classes of compounds usually requires two separate analyses in positive and negative modes, and the optimization of chromatographic conditions [34, 35]. Even when both ionization modes are measured simultaneously (using switching acquisition), the data analysis is usually performed separately for some specific ions and m/z values [36, 37]. In contrast, the possible simultaneous analysis of MS1 and MS2 DIA signals in positive and negative modes, as demonstrated in this work, can increase the number of simultaneously detected compounds with broader chemical coverage and diversity, optimizing efforts in a shorter time. Moreover, the identification steps are extremely facilitated with the direct links established between precursor ions (in MS1) and fragment ions (in MS2 DIA) and with different signals in the two ionization modes. The suitability of the ROIMCR concept associated with the bilinear factor decomposition of both MS1 and MS2 signals arranged in a single matrix has been recently shown by Pérez-López et al. [20], which is extended now in this work with the inclusion of MS1 and MS2 signals in both ionization modes.

In addition, the comparison of ROIMCR results with those obtained using the MS-DIAL method [14, 38, 39] has confirmed the identity of the seventeen amino acids and the two internal standards, with their corresponding m/z values in MS1 and MS2 DIA and with matched retention times. Since MS-DIAL does not allow performing the simultaneous analysis of the positive and negative ionization modes, it was necessary to perform the ion search separately. For the positive mode, MS-DIAL identified the presence of thirteen amino acids detected in the fish embryo samples (reference matched, total score > 850) and the presence of l-threonine and l-aspartate as suggested ions (only MS1 spectra confirmed). On the negative mode, fewer amino acids were identified as reference-matched ions (both MS1 and MS2 spectra confirmed with databank). MS-DIAL only detected five amino acids with a good reference match, and other eight ions were suggested. For both positive and negative modes, the surrogate methionine sulfone was also found as a reference-matched metabolite as well as the internal standard PIPES as a suggested ion. Although MS-DIAL is a well-recognized method that greatly facilitates compound searching, it requires the separated analysis of the MS signals in the two ionization modes.

## Conclusions

Results obtained in this study showed for the first time the suitability of the simultaneous data analysis of MS1( +)/MS2( +)/MS1(-)/MS2(-) signals using the ROIMCR approach in the analysis of a standard mixture of amino acids in solution and fish embryo extracts. Comparison of results obtained in the simultaneous analysis of all acquisition (MS1 and MS2) and ionization (positive and negative) data modes with the results obtained in the individual separate analysis of the different acquisition and ionization modes showed that time efforts and metabolite coverage can be improved in the simultaneous analysis. The link established between the different MS1 + , MS2 + , MS1, and MS2- acquisitions and ionizations for the same chemical compound greatly facilitates the identification of the metabolites present in the analyzed samples. The identification of the amino acids in the fish embryo samples by the proposed ROIMCR method has confirmed the reliability of the proposed strategy for untargeted metabolomics analysis. The approach proposed in this work is an important improvement in the context of the rapid growth of untargeted types of analysis using data-independent acquisition modes. This study encourages further metabolomic investigations using the ROIMCR-described approach combining different MS ionization and acquisition modes.

### Supplementary Information

Below is the link to the electronic supplementary material.

Supplementary file1 (DOCX 713 KB)
