# Supplementary material for: Linking MS1 and MS2 signals in positive and negative modes of LC-HRMS in untargeted metabolomics using the ROIMCR approach
Source: Anal Bioanal Chem. 2023 Aug 17;415(25):6213–25. doi: 10.1007/s00216-023-04893-3 (PMC10558381; doi:10.1007/s00216-023-04893-3)

**Supporting Information**

**Title:** ***Linking MS1 and MS2 signals in positive and negative modes of LC-HRMS in untargeted metabolomics using the ROIMCR approach***

Flávia Yoshie Yamamoto^ab^, Carlos Pérez-López^a^, Ana López-Antia^a^, Silvia Lacorte^a^, Denis Moledo de Souza Abessa^b^, Romà Tauler^a*^

^a -^ Department of Environmental Chemistry, IDAEA-CSIC, Barcelona, Spain

^b –^ Institute of Biosciences, São Paulo State University, São Vicente, Brazil.

***^*^Corresponding author- Romà Tauler -*** [***roma.tauler@idaea.csic.es***](mailto:roma.tauler@idaea.csic.es-)

***Department of Environmental Chemistry, IDAEA-CSIC***

***Jordi Girona, 18-26, 08034 Barcelona, Spain***

1. ***Metabolites extraction in fish embryo samples***

The viable fish embryos (approximately 120 per beaker, 5 replicates) were maintained in 200 ml of reconstituted water (CaCl_2_ 294.0 mg L^-1^, MgSO4 123.3 mg L^-1^, KCl 5.7 mg L^-1,^ and NaHCO3 64.7 mg L^-1^) for 96 hours, with the exchange of half of the solution every 24 h according to OECD 203 guidelines [27]. The surviving individuals (>82%) were frozen at -80^o^C until the analysis and were transported to the Institute of Environmental Assessment and Water Research (Barcelona, Spain) under dry ice (<-20^o^ C).

For the extraction, the samples were previously homogenized with 900 µL of ice-cold methanol (Fisher) by vortexing for 15 s and in the Tissue lyzer LT (Qiagen) for 4 min (40 Hz). The clean-up consisted in adding 500 µL of MilliQ water and 400 µL of chloroform to separate metabolites (upper phase) from unwanted lipids (lower phase). The aqueous fraction containing the metabolites, including amino acids, was evaporated under nitrogen gas flow and reconstituted in 450 µL of 1:1 acetonitrile: water (v/v).

1. ***Parameters for Liquid Chromatography***

Chromatographic parameters selected for the separation of the metabolites using an Acquity UPLC BEH HILIC column are described in Table S1. The protocol was adapted from Waters Protocol, with few modifications (eg. flow rate, injection volume, column temperature and elution gradient time). The mobile phase consisted of A (50:50 Acetonitrile/Water, 10 mM ammonium formate + 0.2% formic acid) and B (90:10 Acetonitrile/Water, 10 mM ammonium formate + 0.2% formic acid). The gradient of elution started with 99.9% of B1 increasing to 75% in 4 min and 50% in 6 min. Concentrations of B1 reached 10% in 7 min and returned to 99.9% after 8 min until 10 min.

Table S1. Chromatographic Parameters used for the separation of the metabolites, both for the amino acids standard solution and the fish embryo samples.

| **Experimental Conditions for Chromatographic Separation** | | |
| --- | --- | --- |
|  | Waters Protocol | New Protocol |
| Column | ACQUITY UPLC BEH HILIC, 2.1 x 50 mm, 1.7 μm | ACQUITY UPLC BEH HILIC, 2.1 x 50 mm, 1.7 μm |
| Mobile Phase A: | 10 mM NH4COOH, 0.2% HCOOH in 50:50 ACN:  H2O | 10 mM NH4COOH, 0.2% HCOOH in 50:50 ACN:  H2O |
| Mobile Phase B: | 10 mM NH4COOH, 0.2% HCOOH in 90:10 ACN:  H2O | 10 mM NH4COOH, 0.2% HCOOH in 90:10 ACN:  H2O |
| Flow Rate: | 0.529 mL/min | 0.4 mL/min |
| Injection Volume: | 5.0 μL | 2.0 μL |
| Sample Diluent: | 73:25:2 ACN:MeOH:H2O with 0.2% HCOOH and  5 μM HCl | 50:50 ACN:  H2O |
| Temperature: | 30 C | 50 C |
| Instrument: | Waters ACQUITY UPLC with SQ Mass Detector | Bruker Impact II QTOF |
| Sample Concentration: | 5 μg/mL | 2 μg/mL |
|  |  |  |
| **Elution Gradient (New Protocol)** | | |
| **Time (min)** | **% A1** | **% B1** |
| **0** | 0.1 | 99.9 |
| **4** | 25 | 75 |
| **6** | 50 | 50 |
| **7** | 90 | 10 |
| **8** | 0.1 | 99.9 |
| **10** | 0.1 | 99.9 |

1. ***Parameters for Mass Spectrometry***

Data independent acquisition parameters selected for the detection of the metabolites by the QTOF(Bruker Impact II) analyzer using the full scan broad-band collision-induce dissociation mode are described in the Table S2.

Table S2. Mass Spectrometry Parameters for the detection of the metabolites in the amino acids standard and the fish embryo samples. Similar conditions were considered for the positive and negative modes of ionization, except that for the negative mode the normalized collision energy was higher for the negative ionization.

| Full scan broad-band collision-induced dissociation (bbCID) | | |
| --- | --- | --- |
|  | **Positive** | **Negative** |
| Mass range (m/z) | 60 to 1000 | |
| End Plate | 500V | 500V |
| Capillary | 2500 V | 2500 V |
| Nebulizer | 2.0 Bar | 2.0 Bar |
| Dry Gas | 8.0 L.min^-1^ | 8.0 L.min^-1^ |
| Dry Temperature | 200^o C^ | 200^o C^ |
| AIF: normalized collision energy (NCE) | 20 <-> 30 eV (alternating) | 24 <-> 36 eV (alternating) |
| Ion energy | 4 eV | 6 eV |
| Prepulse storage | 6 µs | 4 µs |

1. ***Regions of interest (ROI)***

The implementation of the ROI procedure filters the signals of the datasets, avoiding background noise and selects those more relevant according to a set of three parameters: 1) an MS signal intensity threshold to disregard instrumental noise; 2) a mass deviation related to the mass resolution of the used mass spectrometer to distinguish among different m/z values; 3) a minimum number of MS signals that define a chromatographic peak depending on the LC system used and on the MS detector speed.

A lower signal intensity threshold (eg. 0.1% of maximum intensity) may consider more m/z values allowing the detection of more compounds at lower concentrations or with poorer ionization efficiencies. On the other hand, a too-low intensity threshold (eg. <0.1% of maximum intensity) could include a significant amount of experimental noise in the final results, hampering the distinction between noise and analyte signals with poor ionization or low concentrations.

1. ***MCR-ALS analysis***

The application of the MCR-ALS to all these MSROI data matrices followed the same steps:

i) Initial estimation of the number of components (N) to be resolved. This includes chemical components as well as possible other signal contributions from the solvents, chromatographic column, or spectrometer signals, including baseline and background. These different contributions are usually discerned from the features of their concentration and spectral profiles in **C** and **S^T^** factor matrices. Initially, the number of components proposed can be selected visually from the plot of sizes of the singular values (or eigenvalues) of the data. MCR-ALS can be then tested with a different number of components and select the model which better explains the data without overfitting nor including extra noisy components without chemical meaning.

ii) Initial estimation of one of the two-factor matrices, either C or S^T^. This can be performed by selecting the N more different MS spectra at the different retention times (in the rows of the data matrix **D_MS_**) or the more different chromatograms at the different ROI m/z values (in the columns of the data matrix **D_MS_**). A simple way to perform this operation is with the purest variable selection used in the SIMPLISMA method (Windig 1991; Bogomolov & Hachey, 2007; Nardecchia & Duponchel, 2020; ).

iii) MCR-ALS optimizes **C** and **S^T^** factor matrices using an alternating least squares optimization, in two successive steps expressed in the two minimization Equations 6 and 7

$\min_{\hat{C},const}\left\| {\hat{\mathbf{D}}}_{\mathbf{PCA}}-\hat{\mathbf{C}} {\hat{\mathbf{S}}}^{\mathbf{T}} \right\|$ Equation 6

$\underset{\hat{S}^{T},const}{\mathrm{Min}}\left\| {\hat{\mathbf{D}}}_{\mathbf{PCA}}-\hat{\mathbf{C}} {\hat{\mathbf{S}}}^{\mathbf{T}} \right\|$ Equation 7

Where **D_PCA_** refers to the ROIMS data matrix reproduced for the number of components selected in step and $\hat{\mathbf{C}}\mathbf{and}{\hat{\mathbf{S}}}^{\mathbf{T}}$ are the current estimations of **C** and **S^T^.** During the optimization, a set of constraints are applied, which in the case of the LC/Q-TOF data investigated in this work were only the non-negativity of the elution and mass spectra profiles in **C** and **S^T^** factor matrices, and the normalization of the mass spectra profiles in **S^T^** to have maximum intensity values of one. This ALS optimization ends when convergence is achieved or sets a limiting number of iterations. The quality of the results can be evaluated by the amount of experimental variance (R^2^, Equation 8) and in case of validation and compound identification, the recovery of the correct profiles can be estimated by the similarity between the theoretical mass spectra profiles and their estimations by the ROIMCR method, using, for instance, their pair-wise correlation coefficient, r^2^, or the angle between the two spectral vectors in Equations 9 and 10

$R^{2}=100\times(1-\frac{\sum_{i=1}^{m} \sum_{j=1}^{n} \left( d_{\mathrm{ij}}-\hat{d}_{ij} \right)^{2}}{\sum_{i=1}^{m} \sum_{j=1}^{n} d_{\mathrm{ij}}^{2}} )$ Equation 8

$r^{2}= \frac{\mathbf{x}\mathbf{y}^{T}}{\left\| \mathbf{x} \right\|\left\| \mathbf{y} \right\|}$ Equation 9

$angle=180\frac{180}{\pi}\times arccos\left( \frac{\mathbf{x}\mathbf{y}^{T}}{\left\| \mathbf{x} \right\|\left\| \mathbf{y} \right\|} \right)$ Equation 10

Computer programs and software for the ROIMCR and MCR-ALS methods are freely available as a graphical-user-friendly interface (GUI) in the MATLAB® environment (Jaumot et al., 2015; Pérez-Cova et al., (2021))

1. ***MS-DIAL parameters for the identification of the metabolites***

The identification of the metabolites was confirmed using the MS-DIAL according to the parameters described in the Table S3.

Table S3. MS-DIAL parameters used for the confirmation of the metabolite’s identification in the amino acid’s standard solution for both positive and negative ionization modes. The analysis for the identification of amino acids in each ionization mode was performed independently.

| **MS-DIAL Parameters** | |
| --- | --- |
| Ionization type | Soft ionization |
| Separation type | Chromatography |
| MS Method type | All-ions with multiple CEs |
| Data type | Centroid data |
| Ion mode | Positive/Negative separated |
| Mass error tolerance | 0.01 |
| RT begin | 0.5 min |
| RT end | 8 min |
| Mass range | 60-1000 |
| Minimum peak height | 1,000 (~ 0.05%) |
| Mass slice width | 0.1 |
| Smoothing level | 3 scan |
| Minimum peak width | 5 scan |
| Sigma window value | 0.5 |
| RT tolerance | 1 min |

1. **ROIMCR Results**

For instance, the original raw data files of the amino acids’ standard (.cdf) of 398.617 MB in the positive mode and 114.288 MB for the negative mode were compressed to 213 KB (600 x 1262 ROIs) and 153 KB (600 x 1168ROIs), respectively, after ROI analysis of MS1 and MS2 augmented tables. Similarly, the 7 fish embryo samples of an average of 442.612 MB each (all together occupying 3.098 GB) in the positive mode and 113.922 MB (together 797.458 MB) in the negative mode, were reduced to 297 KB and 153 KB, respectively.

Figure S1. Total Ion Chromatogram (TIC) of the amino acids standards obtained by the LC-HRMS in positive and negative ionization mode of acquisition (separated) containing both the MS1 and MS2 datasets already arranged in the same data matrix.


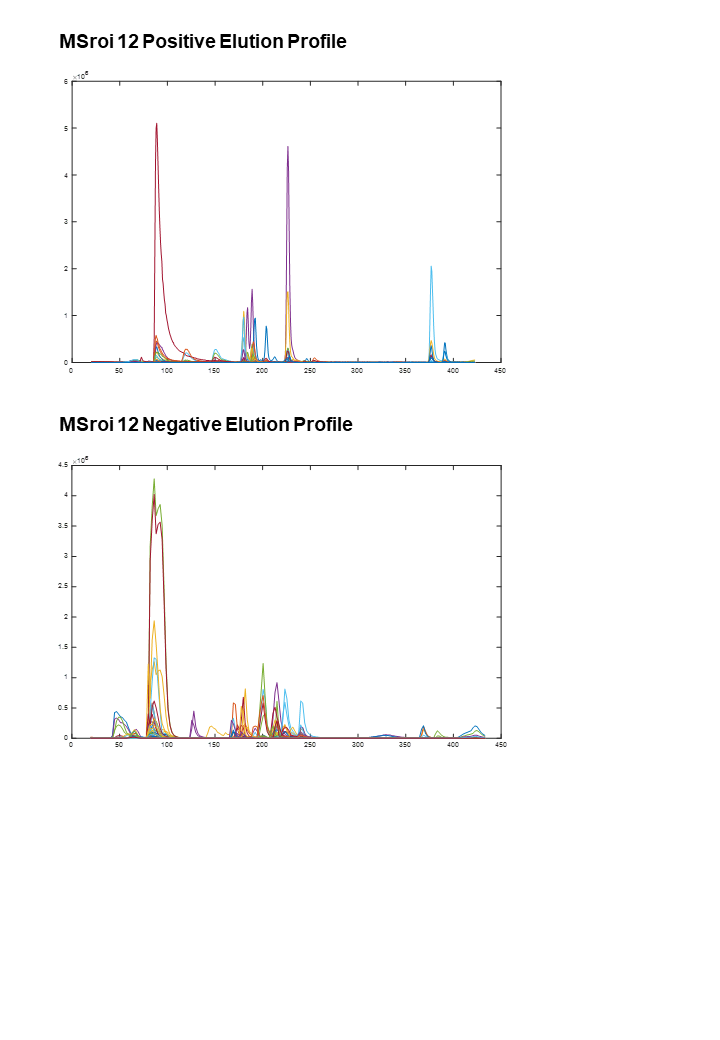


Table S4. Summary of ROIMCR results of the analysis of the amino acids’ standard and the fish embryo samples at each acquisition and ionization mode performed separately and simultaneously.

| **Dataset^1^** | **Nr of ROIs^2^** | **Nr of ROIs selected^3^** | **Constraints^4^** | **R2^5^** |  |  | **N components^6^** |
| --- | --- | --- | --- | --- | --- | --- | --- |
| **D_MS1+_** aa | 351 | 76 | Non-negat. | 99.994 |  |  | 30 |
| **D_MS2+_** aa | 911 | 86 | Non-negat. | 99.909 |  |  | 30 |
| **D_MS12+_** aa | 1262 | 162 | Non-negat. | 99.971 |  |  | 30 |
| **D_MS1-_** aa | 445 | 83 | Non-negat. | 99.99 |  |  | 30 |
| **D_MS2-_** aa | 723 | 78 | Non-negat. | 99.936 |  |  | 30 |
| **D_MS12-_** aa | 919 | 161 | Non-negat. | 99.875 |  |  | 30 |
| **D_MS12+-_** aa | 2181 | 323 | Non-negat. | 99.595 |  |  | 30 |
| **D_MS1+_** samp | 1023 | 200 | Non-negat. | 99.997 |  |  | 100 |
| **D_MS2+_** samp | 1100 | 200 | Non-negat. | 99.989 |  |  | 100 |
| **D_MS12+_** samp | 2123 | 400 | Non-negat. | 99.978 | 0.979 | 1.487 | 100 |
| **D_MS1-_** samp | 687 | 115 | Non-negat. | 99.996 | 0.567 | 0.579 | 100 |
| **D_MS2-_** samp | 727 | 74 | Non-negat. | 99.995 | 0.646 | 0.648 | 70 |
| **D_MS12-_** samp | 1414 | 189 | Non-negat. | 99.981 | 1.285 | 1.381 | 100 |
| **D_MS12+-_** samp | 1055 | 589 | Non-negat. | 99.892 | 2.372 | 3.279 | 100 |

Table S5. List of MS2 fragment ions corresponding for each amino acid in the positive and negative modes obtained in the ROIMCR analysis of the amino acid’s mixture standard.

| Aminoacids | mz MS2 [+] | mz MS2 [-] |
| --- | --- | --- |
| Glycine | - | 74,0223; 72,00671 |
| L-Alanine | 90,0543 | 88,0374; 74,02239 |
| L-Serine | 106,0492; 60,0441; 88,0387 | 104,0321; 61,98615; 88,0375 |
| L-Proline | 116,0697; 70,065 | - |
| L-Valine | 118,0853; 72,0802 | - |
| L-Threonine | 120.0653; 70.0646 | 118,0474; 74,02232 |
| L-Isoleucine | 132,1007 |  |
| L-Leucine | 132,1007; 86,096 |  |
| L-Aspartic acid | 134,0434; 84,9591 | 132,031; 88,0376; 114,999 |
| L-Lysine | 147,1116;121,9651 | 145,0939; 137,9106 |
| L-Glutamic acid | 148.0592; 84.0436; 130.0489; 102.0546 | 146,0415; 128,0314; 102,0529 |
| L-Methionine | 150,057; 133,0306; 104,0519 | 148,0397; 112,9824 |
| L-Histidine | 156,0752; 110,0704; 83,0597 | 154,0577; 93,046; 137,0312; 110,069; 80,0356 |
| L-Phenylalanine | 166,0846; 120,0797; 103,0533; 86,09583 | 164,0671; 147,041; 72,0069 |
| L(+)-Arginine | 175,1174; 70,0648; 116,0697; 130,0965; 60,05525; 158,092 | 173,0992; 131,0787; 146,9567 |
| L-Tyrosine | 182,0794; 136,0743; 123,043; 165,053; 91,0535 | 148,0395; 180,0615; 154,943; 130,0838; 163,0355; 119,0468 |
| L-Cystine | - |  |
| Methionine Sulfone | 136,0466 | 180,0284; 78,9835; 63,9604 |
| PIPES | 70,0646; 303,0646; 152,0362; 195,0779 | 301,0446; 193,0597 |

Figure S2. Elution profiles of the of the aminoacids present in the standard mixture sample resolved by MCR-ALS (C matrix in Equation 4) of the LC-HR MS1 MS2 DIA in positive and negative ionization modes analysis.


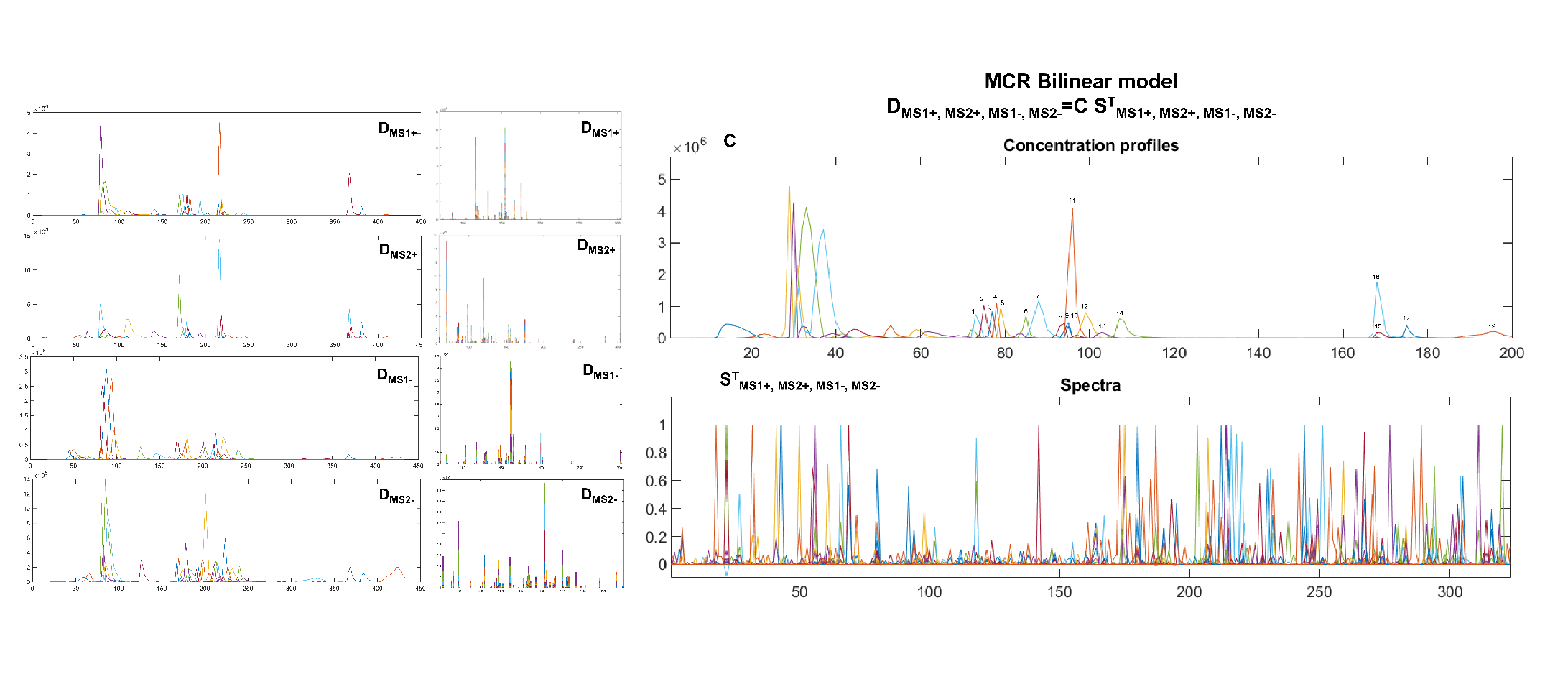


Figure S3. Elution profiles of the of the metabolites present in the fish embryo samples resolved by MCR-ALS (C matrix in Equation 4) of the LC-HR MS1 MS2 DIA in positive and negative ionization modes analysis.


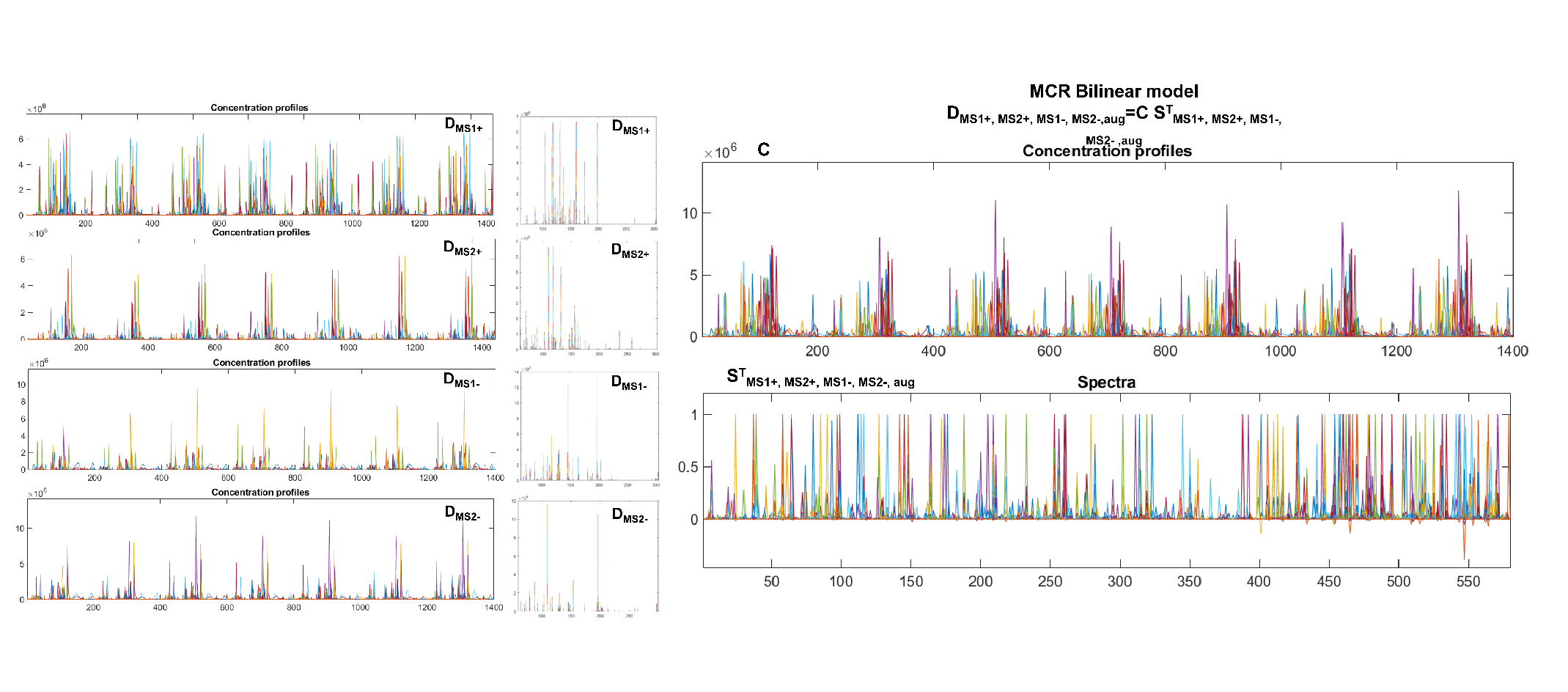

Supplement: Supplementary file 1 — Supplementary file1 (DOCX 713 KB) [file 216_2023_4893_MOESM1_ESM.docx]
